# Fission yeast arrestin-related trafficking adaptor, Arn1/Any1, is ubiquitinated by Pub1 E3 ligase and regulates endocytosis of Cat1 amino acid transporter

**DOI:** 10.1242/bio.20148367

**Published:** 2014-05-29

**Authors:** Akio Nakashima, Shinji Kamada, Fuyuhiko Tamanoi, Ushio Kikkawa

**Affiliations:** 1 Biosignal Research Center, Kobe University, Kobe 657-8501, Japan; 2 Department of Microbiology, Immunology and Molecular Genetics, Molecular Biology Institute, Jonsson Comprehensive Cancer Center, University of California, Los Angeles, CA 90095-1489, USA

**Keywords:** Arrestin-related trafficking adaptor, ART, Arn1/Any1, Amino acid uptake, Endocytosis of transporter, Ubiquitination

## Abstract

The Tsc1–Tsc2 complex homologous to human tuberous sclerosis complex proteins governs amino acid uptake by regulating the expression and intracellular distribution of amino acid transporters in *Schizosaccharomyces pombe*. Here, we performed a genetic screening for molecules that are involved in amino acid uptake and found Arn1 (also known as Any1). Arn1 is homologous to ART1, an arrestin-related trafficking adaptor (ART) in *Saccharomyces cerevisiae*, and contains a conserved arrestin motif, a ubiquitination site, and two PY motifs. Overexpression of *arn1*^+^ confers canavanine resistance on cells, whereas its disruption causes hypersensitivity to canavanine. We also show that Arn1 regulates endocytosis of the Cat1 amino acid transporter. Furthermore, deletion of *arn1*^+^ suppresses a defect of amino acid uptake and the aberrant Cat1 localization in *tsc2*Δ. Arn1 interacts with and is ubiquitinated by the Pub1 ubiquitin ligase, which is necessary to regulate Cat1 endocytosis. Cat1 undergoes ubiquitinations on lysine residues within the N-terminus, which are mediated, in part, by Arn1 to determine Cat1 localization. Correctively, Arn1 is an ART in *S. pombe* and contributes to amino acid uptake through regulating Cat1 endocytosis in which Tsc2 is involved.

## INTRODUCTION

Proper regulation of distribution of plasma membrane proteins in response to environmental cues is important for securing cell growth and survival. In mammalian cells, members of the G-protein-coupled receptor family, which couple primarily to heterotrimeric G-proteins and β-arrestins, initiate a wide range of signaling events involved in several cellular processes when they bind to a diverse array of ligands, such as hormones and peptides (Musnier et al., 2010; Shukla et al., 2011). Desensitization and endocytosis of G-protein-coupled receptors following the agonist binding are mediated by β-arrestins, which function as adaptor molecules that combine E3 ubiquitin ligases with the receptors to ubiquitinate the receptors (Shenoy and Lefkowitz, 2011). β-Arrestins also mediate internalization of growth factor receptors, cytokine receptors, and channels (Lefkowitz et al., 2006).

In the budding yeast *Saccharomyces cerevisiae*, subcellular distribution of several plasma membrane transporters for nutrients including amino acids, nucleobases, and metals is regulated in response to environmental levels of those substrates so that their uptake is regulated. Similar to the regulation of membrane proteins in mammalian cells, ubiquitination of those transporters, which is catalyzed by Rsp5, a HECT-type E3 ubiquitin ligase in the Nedd4 family, governs their intracellular trafficking (Lauwers et al., 2010). It has recently been demonstrated that arrestin-related trafficking adaptors (ARTs) in *S. cerevisiae* containing structurally conserved features with the mammalian arrestin proteins interact with Rsp5 and act as adaptor molecules for the ubiquitination of the nutrient transporters by the ubiquitin ligase to regulate their intracellular trafficking. Moreover, the ARTs are themselves ubiquitinated by Rsp5 (Lin et al., 2008; Nikko et al., 2008; Nikko and Pelham, 2009; O'Donnell et al., 2010; Hatakeyama et al., 2010; Merhi and André, 2012).

In the fission yeast *Schizosaccharomyces pombe*, the Tsc1–Tsc2 complex is a homologous protein complex of human tuberous sclerosis complex (TSC), TSC1 and TSC2, a GTPase-activating protein for the Rheb small GTPase, which is involved in cell growth through the regulation of mechanistic target of rapamycin complex (mTORC1) signaling (Matsumoto et al., 2002; Sengupta et al., 2010). The Tsc1–Tsc2 complex and its target protein, Rhb1, the ortholog of human Rheb, engage in incorporation of amino acids and nucleobases by regulating the localization and expression of the nutrient transporters (Matsumoto et al., 2002; van Slegtenhorst et al., 2004; Urano et al., 2005; Nakase et al., 2006; Aspuria and Tamanoi, 2008; Murai et al., 2009). In addition, like the mammalian system, the Tsc1–Tsc2 complex and Rhb1 are also involved in the TORC1 signaling that plays a critical role in the switch between cell proliferation and differentiation in response to nutrient availability and controls the phosphorylation and activity of Psk1, an ortholog of p70 S6 kinase, which, in turn, regulates the phosphorylation of ribosomal protein S6 (Urano et al., 2005; Alvarez and Moreno, 2006; Uritani et al., 2006; Hayashi et al., 2007; Matsuo et al., 2007; Urano et al., 2007; Weisman et al., 2007; Nakashima et al., 2010; Nakashima et al., 2012).

Pub1 is a homolog of Rsp5 ubiquitin ligase in *S. pombe* and has been identified as a ubiquitin ligase that ubiquitinates and downregulates Cdc25, which is a tyrosine phosphatase for the Cdc2 cyclin-dependent kinase (Nefsky and Beach, 1996). On the other hand, Pub1 is involved in cell proliferation in media at low pH (Saleki et al., 1997). Furthermore, it also contributes to amino acid uptake via the regulation of the localization of Aat1 and Cat1, transporters for general amino acids and cationic amino acids, respectively (Karagiannis et al., 1999; Aspuria and Tamanoi, 2008; Nakase et al., 2012). Pub1 mediates ubiquitination of Aat1, which governs its subcellular distribution (Nakase et al., 2012). Intriguingly, in addition, loss of Pub1 suppresses mislocalization of Cat1 in the *tsc2* disruptant (Aspuria and Tamanoi, 2008). However, the regulatory mechanism of amino acid uptake in which the Tsc1–Tsc2 complex and Pub1 are involved remains largely unclear. As the TSC molecules are not conserved in *S. cerevisiae* (Aspuria et al., 2007), *S. pombe* is an adequate model organism to gain insight into the regulation of amino acid incorporation and the amino acid transporters by the Tsc1–Tsc2 complex.

In this study, in a genetic screening using an *S. pombe* genomic library, we identified *arn1*^+^ (also known as *any1*^+^) encoding an ART as a molecule whose overexpression led to resistance to canavanine in wild-type cells. Arn1 was involved in the regulation of Cat1 endocytosis and acted downstream of Tsc2 in the regulation of amino acid uptake. Arn1 required the interaction with and ubiquitination by Pub1 for its function. In addition, the ubiquitinations of Cat1 determined its subcellular distribution, which may be mediated, in part, by Arn1. Collectively, these results suggest that Arn1 contributes to amino acid uptake as an ART in *S. pombe* through regulation of endocytosis of Cat1.

## RESULTS

### Identification of *arn1*^+^ that causes resistance to canavanine in a genetic screening

**Fig. 1.**
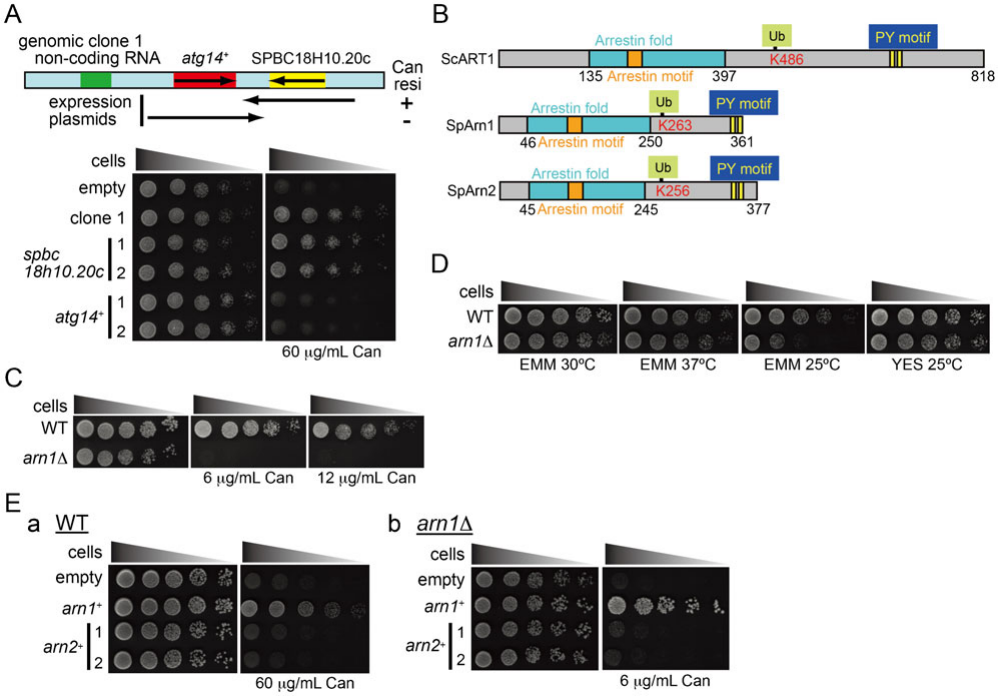
Identification of *arn1*^+^ as a gene that causes resistance to canavanine in a genetic screening. (A) The upper panel shows a schematic representation of the predicted genomic fragment in the clone 1 plasmid. The results in the lower panels are summarized on the right of the diagram (+, −). The lower panels: JUp1211 cells carrying an empty vector, the genomic clone 1 plasmid, pAL-*spbc18h10.20c*, or pAL-*atg14*^+^ were spotted on EMM with or without 60 µg/ml canavanine and incubated for 3 days (EMM) or 5 days (EMM with canavanine). (B) Comparison of the predicted structure of *S. cerevisiae* ART1, *S. pombe* Arn1, and *Sp*Arn2. Each homologous protein contains one arrestin fold domain (blue box), one arrestin motif (orange box), and two PY motifs (yellow boxes) in addition to a conserved lysine residue (red text with yellow–green box). (C) JUp1204 (WT) and AN0196 (*arn1*Δ) were spotted on EMM and EMM with canavanine as indicated and incubated as in panel A. (D) L972 (WT) and AN0195 (*arn1*Δ) were spotted on EMM and YES and incubated for 3 days at temperatures as indicated. (E) JUp1211 (WT) and AN0194 (*arn1*Δ) carrying pAL-KS (empty), pAL-*arn1*^+^, or pAL-*arn2*^+^ were spotted on EMM and EMM with canavanine as indicated and incubated for 3–4 days.

Canavanine, a toxic analog of arginine, is taken up in a manner similar to that of amino acid uptake that utilizes Cat1, a primary cationic amino acid transporter, and consequently causes a severe growth defect of *Schizosaccharomyces pombe* cells (Aspuria and Tamanoi, 2008). To gain insight into the regulation of amino acid uptake, we screened for genes using an *S. pombe* genomic library that suppress the growth defect on a solid medium containing canavanine when they were expressed in the high-copy-number plasmid and obtained two genomic clones, clone 1 and clone 8, from 6×10^4^ transformants. Cells carrying either of the genomic clones showed tolerance to a high concentration of canavanine compared with cells carrying the empty vector (supplementary material Fig. S1A). Sequence analysis revealed that each clone includes distinct chromosomal fragment containing the predicted coding regions and a non-coding RNA as listed in supplementary material Table S1. Because the clone 1 transformant showed more resistance to canavanine than that of clone 8, we looked for the attributes in clone 1 that contribute to the canavanine tolerance. Two predicted open reading frames included in the genomic region of clone 1 are annotated as an ortholog of *atg14*^+^ and SPBC18H10.20c in the *S. pombe* database, PomBase (http://www.pombase.org) (supplementary material Table S1; Fig. 1A). We, thus, first constructed high-copy-expression plasmids in which either of the open reading frames together with its 5′ and 3′ flanking regions is integrated and expressed under its own promoter, and then evaluated the tolerance to canavanine of their transformants (Fig. 1A). Growth of cells expressing the *atg14*^+^ homolog alone on the canavanine-containing medium was inhibited as severely as the control cells transformed with the empty vector, whereas cells expressing SPBC18H10.20c were grown comparably to the clone 1 transformant. Similar results were obtained when these genes were individually overexpressed under the ectopic *nmt1* promoter (Maundrell, 1993) (supplementary material Fig. S1B). These results suggest that overexpression of SPBC18H10.20c confers resistance to canavanine.

Searching the ortholog of the gene product encoded by SPBC18H10.20c using the BLAST program revealed that it exhibits a high homology to ART1, a member of the ART family in *Saccharomyces cerevisiae*, which includes a conserved arrestin motif within an arrestin fold domain, a putative ubiquitination site, and two PY (X_1_–Pro–X_2_–Tyr, where X_1_ is often a Pro) motifs (Lin et al., 2008). It has been known that the arrestin domain in human β-arrestin is required for the interaction with G-protein-coupled receptors (Gurevich et al., 1995; Vishnivetskiy et al., 2004), whereas PY motifs are important to bind to WW (Trp–Trp) domains of HECT-type E3 ubiquitin ligases in the Nedd4 family (Staub et al., 2000; Kay et al., 2000). These structural motifs and the putative modification site are highly conserved in the predicted product of SPBC18H10.20c (Fig. 1B; supplementary material Fig. S2). We therefore named SPBC18H10.20c *arn1*^+^ (*ar*resti*n* 1) and carried out the following experiments. While we were preparing this manuscript, Nakase et al. have independently identified SPBC18H10.20c as a gene whose mutation suppressed a growth defect of *tsc2*Δ caused by a failure of leucine uptake and referred to this gene product as Any1 (Nakase et al., 2013; see Discussion). *S. pombe* possesses one homologous protein to Arn1, which is encoded by SPAC1F12.05, and it shares 38% identity and 57% similarity to Arn1. Similar to Arn1, the homolog is found to contain an arrestin motif, a putative ubiquitination site, and two PY motifs. Therefore, the latter was designated as Arn2 (Fig. 1B).

### Deletion of *arn1*^+^ increases sensitivity to canavanine and suppresses the defect of amino acid uptake in *tsc2*Δ

To elucidate the function of Arn1 in amino acid uptake, we constructed a deletion mutant of *arn1*^+^ and examined its growth on the medium containing canavanine. As opposed to overexpression of *arn1*^+^, its deletion caused higher sensitivity to a low concentration of canavanine than that in the wild type (Fig. 1C). *arn1*Δ also showed slight cold-sensitivity of growth at 25°C on a minimal medium (EMM) but not on a rich medium (YES) (Fig. 1D).

**Fig. 2.**
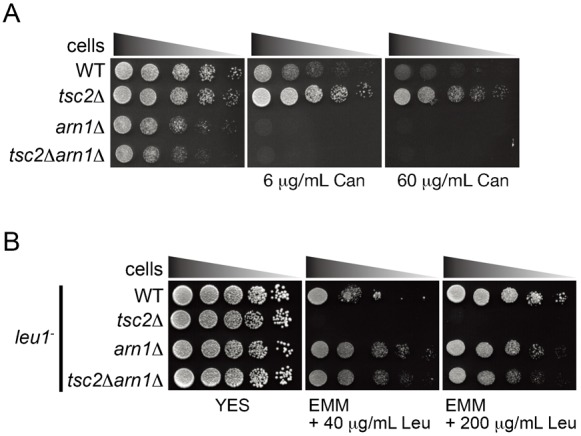
Loss of Arn1 elevates canavanine sensitivity and suppresses the defect of amino acid uptake in *tsc2*Δ. (A) L972 (WT), PJ001 (*tsc2*Δ), AN0196 (*arn1*Δ), and AN0209 (*tsc2*Δ*arn1*Δ) were spotted on EMM and EMM with canavanine as indicated and incubated for 2–4 days. (B) JUp1211 (WT), AN0207 (*tsc2*Δ), AN0194 (*arn1*Δ), and AN0208 (*tsc2*Δ*arn1*Δ) were spotted on YES and EMM with leucine as indicated and incubated for 3 days.

It has been known that a *tsc2* deletion mutant is resistant to a high concentration of canavanine and shows a growth defect in the minimal medium containing leucine when the mutant has a leucine auxotrophy, owing to the defect of amino acid uptake (Matsumoto et al., 2002; van Slegtenhorst et al., 2004). We therefore examined whether Arn1 engages in the Tsc2-dependent amino acid transport. Fig. 2A shows that unlike the *tsc2*Δ single mutant, a double null mutant of *arn1*^+^ and *tsc2*^+^ showed high sensitivity to canavanine as with *arn1*Δ. Furthermore, deletion of *arn1*^+^ in a *tsc2*Δ background suppressed a growth defect of a leucine-requiring *tsc2* mutant on EMM medium containing leucine (Fig. 2B). Taken together, these results suggest that Arn1 participates in the amino acid uptake machinery in which Tsc2 is involved.

### Arn1 is responsible for the endocytosis and stability of the Cat1 transporter

**Fig. 3.**
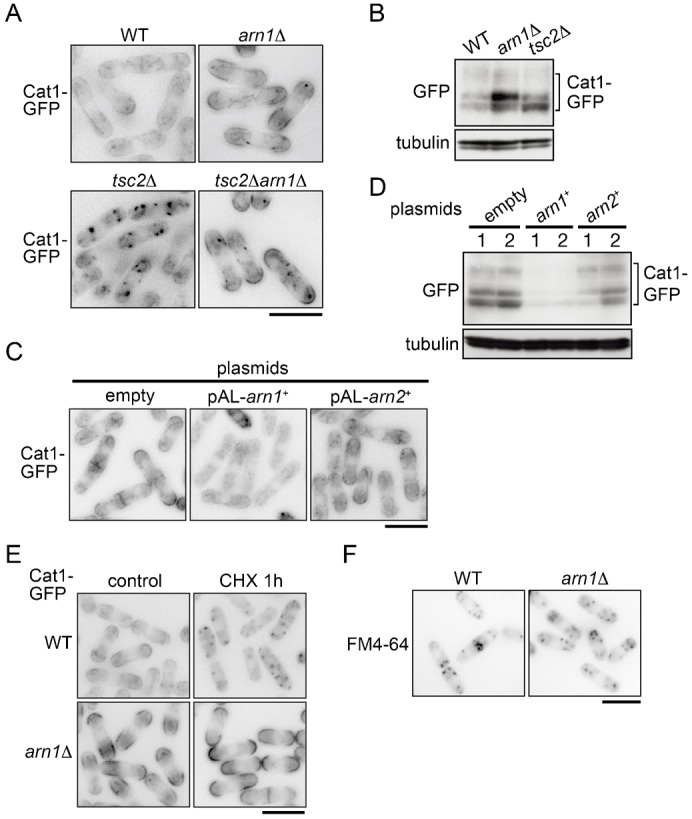
Arn1 participates in the regulation of the subcellular localization and stability of Cat1. (A) PJ390 (WT), AN0223 (*arn1*Δ), PJ380 (*tsc2*Δ), and AN0224 (*tsc2*Δ*arn1*Δ) were grown in EMM. (B) PJ390 (WT), AN0223 (*arn1*Δ), and PJ380 (*tsc2*Δ) were grown in EMM. Tubulin is shown as a loading control. (C) AN0276 carrying the indicated plasmids was grown in EMM. (D) Cells as in panel C were grown in EMM. (B,D) Proteins were probed with the indicated antibodies. (E) PJ390 (WT) and AN0223 (*arn1*Δ) were grown in EMM and then treated with 100 µg/ml cycloheximide for 1 hour. (F) PJ390 (WT) and AN0223 (*arn1*Δ) were grown in EMM and labeled with 50 µM FM4-64 dye on ice for 30 minutes. Five minutes after changing to a fresh medium without the dye at 30°C, the fluorescent signal was observed under a fluorescence microscope. (A,C,E,F) The fluorescence images are shown inverted for clarity. Scale bars: 10 µm.

Tsc2 has been known to regulate the localization of the Cat1 cationic amino acid transporter (Aspuria and Tamanoi, 2008). Thus, the results above raise the possibility that Arn1 regulates the function of Cat1. Possible involvement of Arn1 in the regulation of the subcellular localization of the transporter was examined. In wild-type cells, Cat1-GFP chromosomally expressed under the control of its own promoter was predominantly seen on the plasma membrane (PM) at cell tips and the septum together with punctate cytoplasmic structures (Fig. 3A) (Aspuria and Tamanoi, 2008), whereas the fluorescent signal of Cat1-GFP at these locations was increased in *arn1*Δ (Fig. 3A). Consistent with the results from fluorescent microscopy, the protein level of Cat1-GFP in *arn1*Δ was higher than that in the wild type in western blot analysis (Fig. 3B). On the other hand, Cat1-GFP was mislocalized to and predominantly accumulated in punctate cytoplasmic structures in *tsc2*Δ as previously described (Fig. 3A) (Aspuria and Tamanoi, 2008). Deletion of *arn1*^+^ suppressed the mislocalization of Cat1-GFP in a *tsc2*Δ background, and its distribution in the *tsc2*Δ*arn1*Δ mutant was clearly similar to that in *arn1*Δ (Fig. 3A). The increased PM localization of Cat1-GFP caused by the *arn1* disruption was strongly correlated with the high canavanine sensitivity in *arn1*Δ and *tsc2*Δ*arn1*Δ (Fig. 2A).

In contrast, overexpression of *arn1*^+^ markedly decreased the fluorescent signal of Cat1-GFP (Fig. 3C). In addition, the Cat1-GFP protein in cells overexpressing *arn1*^+^ was hardly detected (Fig. 3D). This Cat1 destabilization by *arn1*^+^ overexpression was correlated with the tolerance to canavanine (Fig. 1A, E; supplementary material Fig. S1B). Taken together, these results suggest that Arn1 is involved in the regulation of the subcellular localization and the protein stability of Cat1.

These findings prompted us to examine whether Arn1 engages in the endocytosis of Cat1. It has been known that cycloheximide induces the internalization of Can1, an arginine transporter, in *S. cerevisiae* (Lin et al., 2008). In wild-type cells, Cat1-GFP was internalized and accumulated in punctate cytoplasmic structures within 1 hour after addition of cycloheximide, whereas Cat1-GFP in *arn1*Δ remained and somewhat increased on the PM and septum even after the cycloheximide addition (Fig. 3E). However, the internalization of FM4-64, a general fluorescent marker of endocytosis, in *arn1*Δ was comparable to that in the wild type (Fig. 3F), suggesting that Arn1 regulates specifically the endocytosis of Cat1.

### Arn2 does not show functional redundancy with Arn1 in the regulation of Cat1

Arn2 is homologous to Arn1 in *S. pombe* as described above, and therefore we examined whether Arn2 is also involved in the regulation of Cat1. As shown in Fig. 1Ea, unlike *arn1*^+^, cells overexpressing *arn2*^+^ were not resistant to canavanine, and overexpression of *arn2*^+^ did not rescue high sensitivity to the arginine analog in *arn1*Δ (Fig. 1Eb). Furthermore, overexpression of *arn2*^+^ provided a little impact on the intracellular distribution and the stability of Cat1-GFP (Fig. 3C,D). These results suggest that Arn2 does not have a redundant function of Arn1 in the control of Cat1.

### Cat1 distribution is regulated in response to nutrient conditions, which is partially dependent on Arn1

**Fig. 4.**
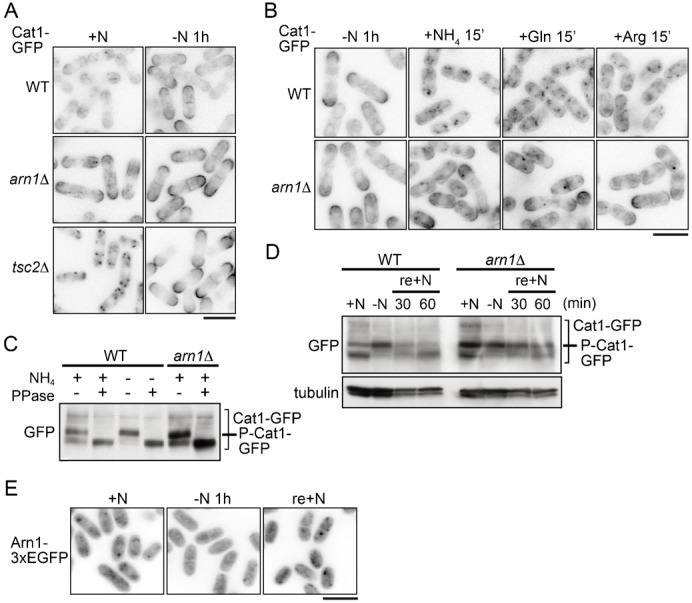
The localization of Cat1 is regulated in response to nutrient conditions, which is partially dependent on Arn1. (A) PJ390 (WT), AN0223 (*arn1*Δ), PJ380 (*tsc2*Δ), and AN0224 (*tsc2*Δ*arn1*Δ) were grown in EMM (+N), washed, and incubated in EMM-N for 1 hour (−N 1 h). (B) PJ390 (WT) and AN0223 (*arn1*Δ) were grown in EMM, washed, and incubated in EMM-N for 1 hour (−N 1 h). Cells were then replaced and incubated in EMM and EMM-N containing glutamine or arginine for 15 minutes. (C) PJ390 (WT) and AN0223 (*arn1*Δ) were grown in EMM. PJ390 was also subsequently incubated in EMM-N for 1 hour. Protein extracts prepared from membrane rich fractions were incubated with or without λ-phosphatase. (D) PJ390 (WT) and AN0223 (*arn1*Δ) were grown in EMM (+N), washed, and incubated in EMM-N for 1 hour (−N). Ammonium was subsequently added to the cultures (final concentration, 0.5%), and cells were incubated for the indicated times (re+N). (C,D) Proteins were probed with the indicated antibodies. (E) AN0352 (Arn1-3×EGFP) was grown in EMM (+N), washed, and incubated in EMM-N for 1 hour (−N 1 h). Ammonium was added to the culture, and cells were incubated for 15 minutes (re-+N). (A,B,E) The GFP images are shown inverted for clarity. Scale bars: 10 µm.

To next examine the effect of nutrient conditions on the distribution of Cat1, cells expressing Cat1-GFP were nitrogen-starved for 1 hour and observed. Nitrogen starvation concentrated Cat1-GFP on the PM in wild-type cells and in *arn1*Δ (Fig. 4A). Its starvation-induced translocation to the PM was also observed in *tsc2*Δ where Cat1-GFP was accumulated in punctate cytoplasmic structures in the nutrient rich medium (Fig. 4A). Furthermore, Cat1-GFP on the PM in the wild type under nitrogen starvation was mostly internalized within 15 minutes following re-addition of nitrogen sources such as glutamine, arginine, and ammonium (Fig. 4B). It is of interest that internalization of Cat1-GFP responded not only to arginine but also to glutamine and ammonium, although Cat1 is a specific transporter for basic amino acids (Aspuria and Tamanoi, 2008). In contrast, in *arn1*Δ, although a portion of the transporter was internalized within 15 minutes following the stimulation with nitrogen sources, the majority remained on the PM (Fig. 4B), suggesting that Arn1 is involved, but is not indispensable, in the nutrient-dependent Cat1 endocytosis.

As shown in Fig. 3B,D, Cat1-GFP was detected as two major bands with minor bands showing low mobility in western blotting analysis, suggesting some posttranslational modifications of Cat1. The electrophoretic mobility shift of the two major Cat1-GFP bands was increased by nitrogen starvation; the lower one disappeared, whereas the upper one was increased (Fig. 4C,D). The λ-phosphatase assay revealed that the mobility shift was due to phosphorylation (Fig. 4C). A similar modification of Cat1 was observed when it was tagged with three tandem HA epitopes (data not shown), suggesting that Cat1 itself is phosphorylated. Fig. 4D shows that increased Cat1 phosphorylation under nitrogen starvation was promptly decreased by re-addition of ammonium. Alteration of Cat1 phosphorylation seems to be correlated with its localization, because when Cat1 was concentrated on the PM by nitrogen starvation, it was highly phosphorylated. Furthermore, Cat1-GFP in *arn1*Δ, which tended to be localized on the PM, showed a higher phosphorylation state than that in the wild type in the nutrient rich medium (Fig. 4D). Consistent results were obtained when cells were treated with cycloheximide. Cat1-GFP in *arn1*Δ following the treatment with cycloheximide, which was accumulated on the PM (Fig. 3E), was highly phosphorylated, whereas it exhibited decreased phosphorylation regardless of nutrient conditions in the wild type or *tsc2*Δ (supplementary material Fig. S3), which was internalized by the treatment (Fig. 3E; data not shown). Collectively, these results suggest that Cat1 is subjected to phosphorylation on the PM in a nutrient-independent manner. Alternatively, the phosphorylation of Cat1 might facilitate its transport to the PM.

To next examine whether Arn1 responds to nutrient conditions, we constructed a strain chromosomally expressing Arn1-3EGFP under its own promoter and initially tested its canavanine sensitivity to assess the function of the fusion protein. Cells expressing Arn1-3EGFP could grow comparably to the control cells in the presence of a low concentration of canavanine, which inhibits the growth of *arn1*Δ (data not shown), suggesting that the fusion protein, at least in part, maintains the function of Arn1. Arn1-3EGFP was distributed throughout the cell, and some were localized in punctate cytoplasmic structures in the growing condition (Fig. 4E). After 1 hour of nitrogen starvation, the fluorescent signals of Arn1-3EGFP on the cytoplasmic puncta were dispersed, and then it was accumulated in the punctate structures again within 15 minutes after refeeding ammonium (Fig. 4E). Accordingly, the localization of Arn1 was regulated in response to nutrient conditions.

### Significance of the conserved motifs and ubiquitination of Arn1 for the regulation of Cat1 endocytosis

**Fig. 5.**
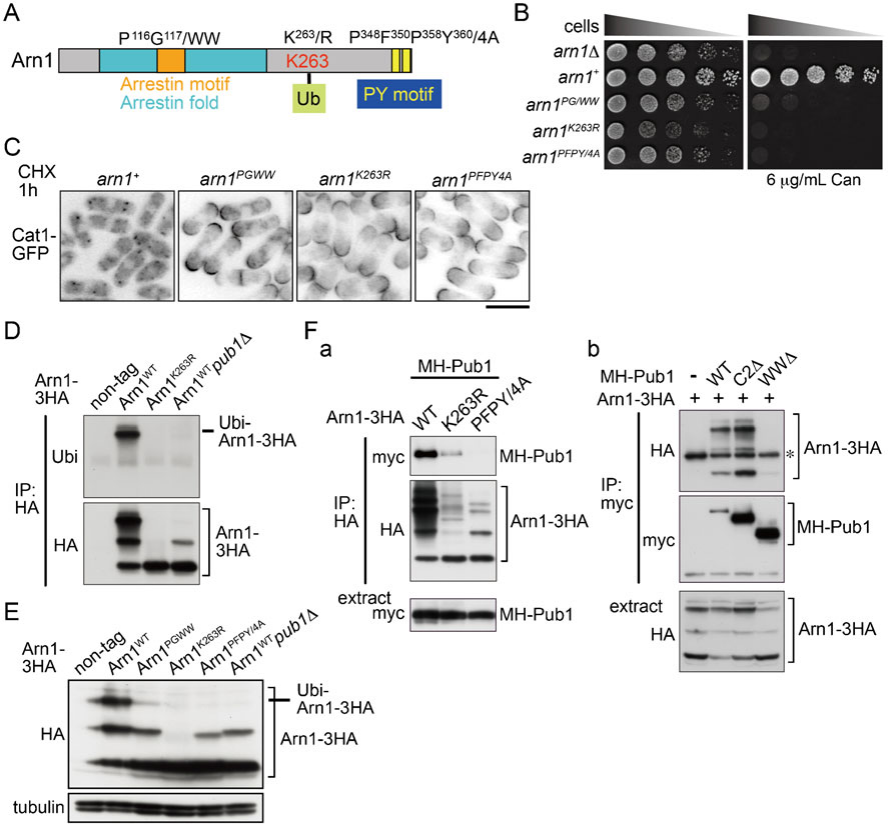
Roles of the conserved motifs and ubiquitination of Arn1 in regulation of Cat1. (A) Schematic diagram of the mutations in Arn1 (P^116^G^117^/WW in an arrestin motif, K^263^/R at a conserved ubiquitination site, and P^348^F^350^P^358^Y^360^/4A in PY motifs). (B) AN0195 (*arn1*Δ), AN0220 (*arn1*^+^), AN0273 (*arn1^PG/WW^*), AN0264 (*arn1^K263R^*), and AN0267 (*arn1^PFPY/4A^*) were spotted on EMM and EMM with 6 µg/ml canavanine, and incubated for 3 and 6 days, respectively. (C) AN0282 (*arn1*^+^), AN0286 (*arn1^PG/WW^*), AN0288 (*arn1^K263R^*), and AN0284 (*arn1^PFPY/4A^*) were grown in EMM and then treated with 30 µg/ml cycloheximide for 1 hour. The GFP images are shown inverted for clarity. (D) L972 (non-tag), AN0220 (Arn1^WT^), AN0264 (Arn1^K263R^), and AN0259 (Arn1^WT^*pub1*Δ) were grown in YES. Arn1-3HA was immunoprecipitated from protein extracts. (E) L972 (non-tag), AN0220 (Arn1^WT^), AN0273 (Arn1^PG/WW^), AN0264 (Arn1^K263R^), AN0267 (Arn1^PFPY/4A^), and AN0259 (Arn1^WT^*pub1*Δ) were grown in YES. (Fa) AN0293 (WT), AN0295 (K263R), and AN0297 (PFPY/4A) strains carrying pREP41-*2myc*-*6His*-*pub1^+^* were grown in EMM. (Fb) AN0293 (Arn1-3HA) carrying an empty vector (−), pREP41-*2myc*-*6His*-*pub1^+^* (WT), -*pub1C2Δ* (C2Δ), or -*pub1WWΔ* (WWΔ) were grown in EMM. Arn1-3HA (Fa) and MH-Pub1 (Fb) were immunoprecipitated from protein extracts. (D,E,Fa,Fb) Proteins were probed with the indicated antibodies. Scale bar: 10 µm.

In *S. cerevisiae*, the arrestin and PY motifs as well as the ubiquitination of ART1 are required for the regulation of membrane protein endocytosis (Lin et al., 2008). Therefore, to address the role of the conserved motifs in Arn1 in the regulation of Cat1, we constructed mutant strains chromosomally expressing 3HA-tagged mutant Arn1 in which the corresponding residues in either the arrestin or PY motifs to those of *S. cerevisiae* ART1 were substituted with tryptophan or alanine (Fig. 5A; supplementary material Fig. S2) and measured their sensitivity to canavanine. As shown in Fig. 5B, either the mutation in the arrestin or PY motifs in Arn1 caused a high sensitivity to a low concentration of canavanine with similar sensitivity as its null mutant, although the Arn1 mutant proteins were expressed similarly to that of the wild type (Fig. 5E). These mutations also caused a defect of the internalization of Cat1 induced by cycloheximide (Fig. 5C), suggesting that the arrestin and PY motifs in Arn1 are important to control Cat1 endocytosis.

Moreover, the substitution of Lys263 of Arn1 to arginine, which corresponds to the Lys486 ubiquitination site in *S. cerevisiae* ART1 (supplementary material Fig. S2; Fig. 5A), also led to the loss of Arn1 function (Fig. 5B,C). This result prompted us to investigate whether Arn1 is actually ubiquitinated. Fig. 5D shows that immunoprecipitated wild-type Arn1-3HA was mainly observed as three bands and that only the top band but not the others was detected with the anti-ubiquitin antibody. In addition, the electrophoretic mobility shifts and the ubiquitination of Arn1-3HA were completely lost by the K263R mutation (Fig. 5D). Taken together, these results suggest that Arn1 is subjected to ubiquitination, which is important in the regulation of Cat1 endocytosis, and that the Lys263 residue directly undergoes or is indirectly involved in its ubiquitination. To next examine the effect of mutations in the arrestin and PY motifs of Arn1 on its ubiquitination, we assessed the ubiquitination of the two Arn1-3HA mutants. Because the PY motif is required for the binding to the WW domains in the NEDD4/Rsp5 ubiquitin ligase (Staub et al., 2000; Kay et al., 2000), as expected, the defect of the PY motifs abolished the top band that corresponded to ubiquitinated Arn1-3HA (Fig. 5D,E), whereas the mutations of the arrestin motif also markedly attenuated its modification (Fig. 5E). These results suggest that the conserved and functional motifs of Arn1, especially the PY motifs, are required for its ubiquitination.

### Pub1 E3 ubiquitin ligase associates with and mediates ubiquitination of Arn1

In *S. pombe*, Pub1 is an ortholog of the Rsp5 HECT-type ubiquitin ligase (Nefsky and Beach, 1996; Saleki et al., 1997) and is involved in trafficking of Cat1 and Aat1 (Aspuria and Tamanoi, 2008; Nakase et al., 2012). Therefore, we examined the involvement of Pub1 in Arn1 ubiquitination and found that deletion of *pub1*^+^ resulted in the defect of Arn1 ubiquitination, which was similar to that observed in the PY motif mutant (Fig. 5D,E). Moreover, as shown in Fig. 5Fa,Fb, 2myc- and 6His-tagged wild-type Pub1 was co-purified with immunoprecipitated wild-type Arn1-3HA, and reciprocally, wild-type Arn1-3HA was bound to immunoprecipitated wild-type 2myc-6His-Pub1. As expected, the PY motifs in Arn1 were necessary for the interaction with Pub1, and the defect of Arn1 ubiquitination caused by the K263R substitution markedly decreased its interaction with Pub1 (Fig. 5Fa). Pub1 contains a C2 domain at the N-terminal and three WW domains in the middle region in addition to a HECT domain at the C-terminal, a catalytic domain of the E3 ubiquitin ligase (supplementary material Fig. S4A) (Nefsky and Beach, 1996; Saleki et al., 1997). Deletion of only the WW domains in Pub1 was sufficient to abolish the interaction with Arn1, whereas deletion of the C2 domain provided little effect on this protein interaction (Fig. 5Fb). Taken together, these results suggest that Pub1 probably directly ubiquitinates Arn1 and that the PY motifs and WW domains mediate the interaction between the substrate and the enzyme as previously reported (Staub et al., 2000; Kay et al., 2000).

It is interesting to note that the middle band of Arn1-3HA was slightly reduced but sufficiently remained in the mutant of the PY motifs or in a *pub1* disruptant, although it was abolished by the K263R substitution (Fig. 5D,E). On the other hand, this band was not detected with the anti-ubiquitin antibody (Fig. 5D). It has been known that in mammalian cells, arrestin-3, which regulates internalization of β_2_-adrenergic receptor, is modified by small ubiquitin-like modifier protein (SUMO) at a lysine residue (Wyatt et al., 2011). Therefore, to explore the possibility that Arn1 undergoes SUMOylation, Arn-3HA was expressed in cells deficient in *pmt3*^+^ encoding the sole SUMO protein in *S. pombe* (Tanaka et al., 1999) or in cells overexpressing 2myc-epitopes tagged Pmt3. The middle band of Arn1-3HA was not abolished by the *pmt3* deletion and did not overlap with 2myc-Pmt3 (data not shown). The additional modification of Arn1, thus, needs to be determined.

### Arn1 and Pub1 cooperatively regulate Cat1 function

**Fig. 6.**
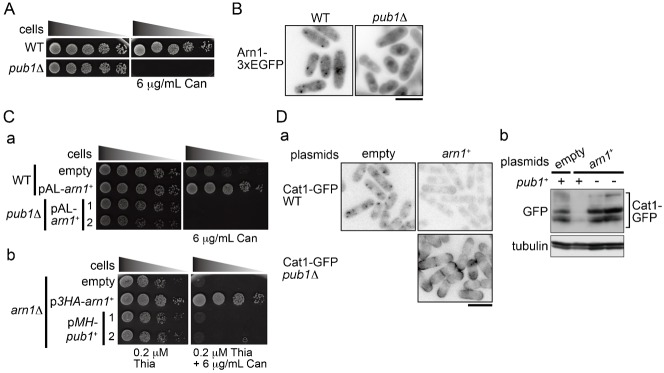
Arn1 and Pub1 cooperatively work on amino acid uptake through Cat1 regulation. (A) L972 and AN0259 (*pub1*Δ) were spotted on EMM and EMM with 6 µg/ml canavanine, and incubated for 4 and 5 days, respectively. (B) AN0351 (WT) and AN0399 (*pub1*Δ) were grown in EMM. (Ca,b) AN276 (WT, a), AN0290 (*pub1*Δ, a), and AN0319 (*arn1*Δ, b) carrying an empty or expression plasmids as indicated were spotted on EMM and EMM with 6 µg/ml canavanine, and incubated for 3 and 4 days, respectively. (Cb) Thiamine was added in media to express moderately *pub1*^+^ to avoid a growth defect caused by overexpression of *pub1*^+^. (Da,b) Strains as in panel Ca were grown in EMM. (Db) Proteins were probed with the indicated antibodies. (B,Da) The GFP images are shown inverted for clarity. Scale bars: 10 µm.

As we reported previously (Aspuria and Tamanoi, 2008), loss of Pub1 led to canavanine hypersensitivity and to stabilization and accumulation of Cat1 to the PM (Fig. 6A; supplementary material Fig. S4B,C). These phenomena were similar to those in *arn1*Δ. Therefore, the genetic relationship between *arn1*^+^ and *pub1*^+^ was next examined. Fig. 6B shows that deletion of *pub1*^+^ dispersed the cytoplasmic punctate Arn1-3EGFP and slightly increased its nuclear accumulation. In addition, the *pub1* deletion completely suppressed tolerance to canavanine and destabilization of Cat1-GFP caused by *arn1*^+^ overexpression (Fig. 6Ca,Da,Db). In contrast, overexpression of *pub1*^+^ did not rescue hypersensitivity of *arn1*Δ to canavanine (Fig. 6Cb). These results suggest that Arn1 and Pub1 cooperatively control amino acid uptake through the Cat1 regulation.

### Cat1 undergoes ubiquitination on the several lysine residues in the N-terminal region, which determine its subcellular distribution

**Fig. 7.**
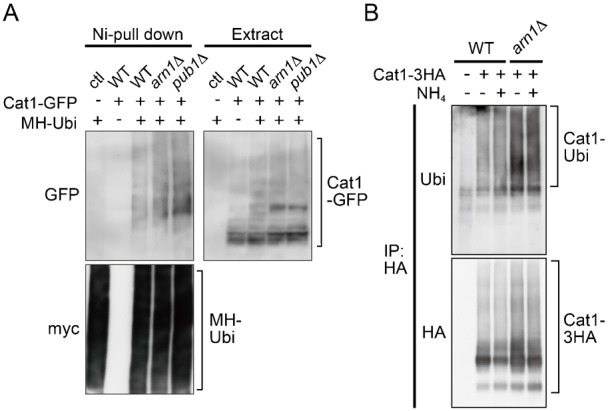
Ubiquitination of Cat1 in *arn1*Δ and *pub1*Δ. (A) JUp1211(ctl), AN0276 (WT), AN0319 (*arn1*Δ), and AN0290 (*pub1*Δ) carrying pREP273 (−) or pREP41-2myc-6His-ubiquitin (+) were grown in EMM. MH-ubiquitin was pulled down from protein extracts. (B) L968 (WT, −), AN0330 (WT, +), and AN0335 (*arn1*Δ) were grown in EMM, washed, and incubated in EMM-N for 45 minutes (−). Cells were further incubated for 15 minutes following the addition of ammonium (+). Cat1-3HA was immunoprecipitated from protein extracts. (A,B) Proteins were probed with the indicated antibodies.

The above results raise the possibility that Arn1 acts as an adaptor molecule for Pub1 to mediate ubiquitination of Cat1 as ART1 in *S. cerevisiae* (Lin et al., 2008). To explore this possibility, we examined ubiquitination of Cat1 in the presence or absence of *arn1*^+^ gene. As shown in Fig. 7A, the pull-down assay using Ni^2+^ resin revealed that the conjugation of Cat1-GFP and 2myc- and 6His-tagged ubiquitin which was exogenously expressed was observed not only in the wild type but also in *arn1*Δ and *pub1*Δ. The Cat1-GFP protein pulled down with ubiquitin was to some extent increased in both mutants (Fig. 7A), but this seems to be due to an increased stability of Cat1 caused by loss of Arn1 or Pub1 as shown in Fig. 3B and supplementary material Fig. S4C. Furthermore, the conjugation of Cat1 with endogenous ubiquitin with or without stimulation by ammonium in *arn1*Δ as well as that in the wild type was also detected when Cat1-3HA was immunoprecipitated (Fig. 7B). A possible role of Arn1 in Cat1 ubiquitination is mentioned in Discussion.

We next attempted to determine the interaction of Arn1 with Cat1 in cells expressing Arn1-3HA and Cat1-GFP under stimulation of glutamic acid as a nitrogen source in the presence of chemical cross linkers, dithiobis (succinimidylpropionate) (DSP) or both DSP and dimethyl 3,3′-dithiobis propionimidate dihydrochloride (DTBP). However, in the conditions we employed, the interaction between Arn1 and Cat1 was not detected (data not shown).

**Fig. 8.**
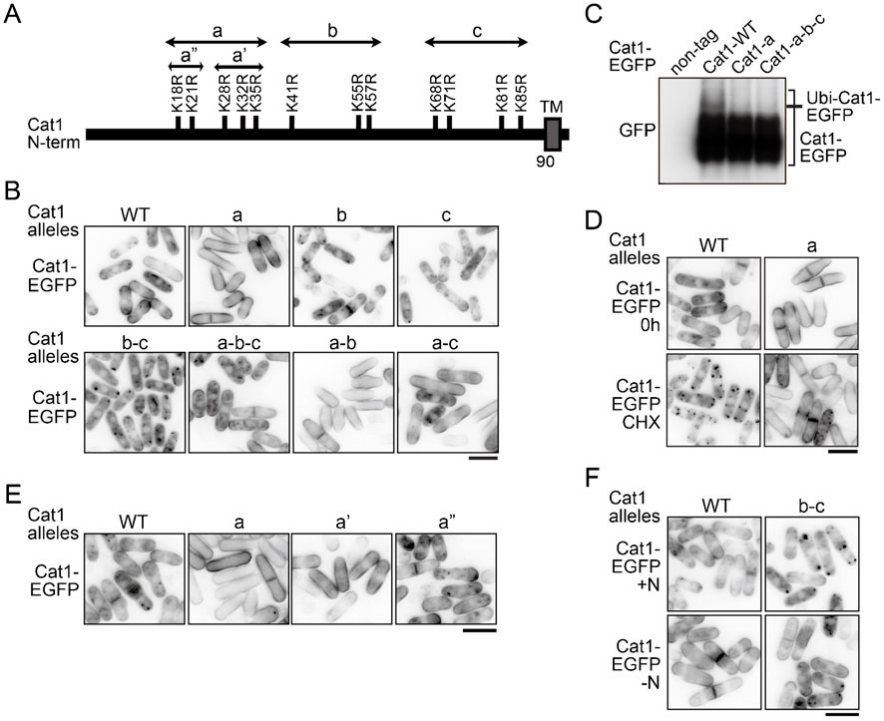
Determination of Cat1 distribution by its modification at the N-terminal lysine residues. (A) Schematic representation of substitutions of lysine residues by arginine in the N-terminal of Cat1. Double-head arrows show the extent of the respective Cat1 mutants including the substitutions of lysine residues. TM denotes a transmembrane domain. (B) JUp1209 cells carrying pREP273-Cat1-WT or the mutants fused with EGFP as indicates were grown in EMM + uracil. (C) L968 (non-tag), AN0541 (Cat1-WT), AN0542(Cat1-a), and AN0543(Cat1-a-b-c) were grown in EMM. Protein extracts were prepared from membrane rich fractions, and proteins were probed with the GFP antibody. (D) JUp1209 carrying pREP273-Cat1-WT or -a-EGFP was grown in EMM + uracil (0 h) and then treated with 30 µg/ml cycloheximide for 1 hour (CHX). (E) JUp1209 carrying pREP273-Cat1-WT or the mutants as indicates were grown in EMM + uracil. (F) JUp1209 carrying pREP273-Cat1-WT- or -b-c-EGFP was grown in EMM + uracil (+N), washed, and incubated in EMM-N for 1 hour (−N). (B,D–F) The GFP images are shown inverted for clarity. Scale bars: 10 µm.

It has been reported that the Aat1 transporter has several potential ubiquitination sites on lysine residues in the cytoplasmic N-terminal region and that those ubiquitinations appear to determine its translocation (Nakase et al., 2012). Cat1 also includes many lysine residues in the region between the N-terminal end and the first transmembrane domain (Fig. 8A). To investigate the effect of the modification of these lysine residues on the localization of Cat1, the Cat1-EGFP mutants in which the lysine residues in the N-terminal region were substituted by arginine as shown in Fig. 8A were expressed, and their localization was examined. As shown in Fig. 8B, the Cat1-a-EGFP mutant was exclusively localized on the PM, whereas either the b- or c-mutant showed little effect on the localization of Cat1-EGFP or those combinations with Cat1-a-EGFP. The combination of b- and c-mutations resulted in predominantly internalized Cat1-EGFP, whereas the all a-b-c-mutations exhibited an intermediate effect between the a- and b-c-mutations on the distribution. The ubiquitination of the Cat1-a- and -a-b-c-EGFP mutants was significantly decreased compared with that of the wild type (Fig. 8C), suggesting that Cat1 undergoes ubiquitination on the lysine residues, at least in part, in the N-terminal region. Furthermore, the Cat1-a mutation notably blocked the internalization of Cat1-EGFP by the cycloheximide treatment, similar to that observed in *arn1*Δ (Fig. 3E, Fig. 8D). Within the Cat1-a mutation, the substitution of the latter 3 lysine residues (Cat1-a′) was enough for its PM localization, whereas the substitution of the former 2 residues (Cat1-a″) showed little effect, which was indistinguishable from that of the wild type (Fig. 8E). On the other hand, nitrogen starvation moved Cat1-b-c-EGFP from punctate cytoplasmic structures to the PM (Fig. 8F). The distribution of Cat1-b-c-EGFP was similar to that of Cat1-GFP in *tsc2*Δ (compare with Fig. 4A). Taken together, these results suggest that modifications of specific lysine residues in the N-terminus of Cat1 by ubiquitin determine its localization.

## DISCUSSION

In mammalian cells, one of the major functions of β-arrestin is to act as an adaptor of E3 ubiquitin ligases, such as MDM2 and NEDD4 that mediate ligand-induced ubiquitination and internalization of G-protein-coupled receptors and the other types of membrane proteins (Lefkowitz et al., 2006; Shenoy and Lefkowitz, 2011). β-Arrestin itself undergoes ubiquitination by Mdm2 (Shenoy et al., 2001). Here, in *S. pombe*, we identified Arn1, a homolog of budding yeast arrestin-related trafficking adaptor (ART) 1, in a genetic screening using an *S. pombe* genomic library for molecules that confer resistance to canavanine, a toxic analog of arginine, when they are highly expressed in wild-type cells. This screening can isolate molecules that participate in amino acid uptake (Fig. 1). We further demonstrated that Arn1 is implicated in endocytosis of Cat1, a primary cationic amino acid transporter, in response to extracellular conditions, such as nutrients and cycloheximide treatment (Fig. 3, Fig. 4B). On the other hand, deletion of *arn1*^+^ rescues a defect of amino acid uptake and the aberrantly internalized Cat1 localization in *tsc2*Δ (Fig. 2). It has been known that the internalized Cat1 in *tsc2*Δ causes the resistance to canavanine (Aspuria and Tamanoi, 2008). The restoration of Cat1 localization in *tsc2*Δ by deletion of *arn1*^+^ abolished the canavanine resistance. These results suggest that Arn1 functions downstream of Tsc2 in amino acid uptake. The TSC molecules are conserved between fission yeast and mammal, whereas there are no counterparts of TSCs in *S. cerevisiae* (Aspuria et al., 2007). Therefore, the finding that the TSC–ART pathway contributes the regulation of membrane transporter distribution in *S. pombe* may provide insight into the relationship between the TSC and arrestin proteins in higher eukaryotes.

Arn1 is predicted to contain one arrestin motif and two PY motifs (Fig. 1B). These motifs in Arn1 were required for the regulation of Cat1 endocytosis (Fig. 5). In addition, Arn1 directly interacted with and was ubiquitinated probably at a conserved lysine residue (Lys263) by Pub1, an E3 ubiquitin ligase in the Nedd4 family. These actions were mediated by the association of the PY motifs in the substrate with the WW domains in the ligase. This ubiquitination in Arn1 was also required for the regulation of Cat1 endocytosis (Fig. 5). Furthermore, epistatic analyses showed that Arn1 and Pub1 cooperatively engaged in Cat1 function (Fig. 6). These findings propose Arn1 as an ART in *S. pombe*, and that the modulation of ART function through ubiquitination by the E3 ubiquitin ligase is evolutionarily conserved among two distantly related yeasts and mammal.

Cat1 was ubiquitinated at multiple sites on lysine residues, at least in part, in the cytoplasmic N-terminal region (Fig. 7, Fig. 8C). The Cat1-a and, especially, Cat1-a′ mutants (substitution of lysine residues from Lys18 to Lys35 and from Lys28 to Lys35 by arginine, respectively (Fig. 8A)) were exclusively localized on the PM and were deficient in their internalization from the PM induced with cycloheximide, whereas the replacement of lysine residues from Lys41 to Lys85 by arginine (b-c mutation) confined the transporter in punctate cytoplasmic structures (Fig. 8B,D). Therefore, Cat1 might undergo different types of ubiquitin-mediated regulations to determine its subcellular localization: one of them (especially ubiquitinations on Lys28–Lys35) promotes endocytosis, and the other (ubiquitinations on Lys41–Lys85) facilitates transport to the PM. On the other hand, nitrogen starvation translocated the Cat1-b-c mutant from punctate cytoplasmic structures to the PM (Fig. 8F). This observation raises the possibility that if nitrogen starvation attenuates the ubiquitinations on Lys28–Lys35, it may be enough for keeping Cat1 on the PM even though the absence of the ubiquitinations on Lys41–Lys85. Therefore, the ubiquitinations on Lys41–Lys85 in Cat1 may alternatively block the ubiquitinations of Lys28–Lys35 to prevent its endocytosis. Cat1 in *tsc2*Δ was accumulated in punctate structures in the cytoplasm in nutrient rich conditions, but the deletion of *arn1*^+^ or nitrogen starvation facilitated translocation of Cat1 to the PM (Fig. 3A, Fig. 4A). Tsc2 may therefore regulate ubiquitinations on Lys41–Lys85 in Cat1.

Here, we could not obtain direct evidence that Arn1 interacts with and mediates ubiquitination of Cat1. However, Arn1 carries one arrestin motif that is known to be important for the interaction with membrane proteins in mammal (Gurevich et al., 1995; Vishnivetskiy et al., 2004), and this motif was required for Cat1 endocytosis (Fig. 5C). Similarly, the arrestin motif of ART1 in *S. cerevisiae* is also necessary to regulate the endocytosis of its targets (Lin et al., 2008). Therefore, Arn1 possibly interacts with Cat1, but its interaction may be transient and weak or, alternatively, require other unknown protein(s). Moreover, the observation that deletion of *arn1*^+^ caused a defect of Cat1 internalization from the PM induced by cycloheximide was comparable to the endocytosis defect of the Cat1-a mutant, which was probably deficient in ubiquitination on lysine residues from Lys18 to Lys35 (Fig. 3E, Fig. 8D). Therefore, Arn1 seems to participate in Cat1 ubiquitination on lysine residues, at least from Lys18 to Lys35, especially from Lys28 to Lys35, which would contribute to Cat1 endocytosis. On the other hand, Cat1 would be ubiquitinated at several lysine residues in addition to the residues modified by Arn1 and Pub1 (Fig. 8). Therefore, as shown in Fig. 7, the defect of ubiquitination caused by loss of Arn1 or Pub1 may be eclipsed by the other multiple ubiquitinations.

As described above, Nakase et al. independently reported that Arn1/Any1 (they named SPBC18H10.20c Any1) regulates subcellular distribution of some transporters including Aat1, an *S. pombe* homolog of *S. cerevisiae* general amino acid transporter Gap1 (Nakase et al., 2010; Nakase et al., 2013). However, Cat1 was not characterized. Although several features of Arn1/Any1 showed in our and their studies are overlapping, some major points are different as summarized: 1) Arn1 and Any1 were identified from distinct screening approaches. We identified *arn1*^+^ as a gene whose high-copy expression confers canavanine resistance on wild-type cells using a *S. pombe* genomic library (Fig. 1), whereas *any1*^+^ was isolated in a screening for mutations that restore a growth defect of *tsc2*Δ owing to a failure of leucine uptake (Nakase et al., 2013). 2) We demonstrated that Arn1 is ubiquitinated probably at Lys263 by Pub1 E3 ubiquitin ligase and that its ubiquitination is important for regulation of Cat1 endocytosis (Fig. 5). This is the first report of the ubiquitination and its function of ART in *S. pombe*. In addition, this study presented the significance of structurally conserved features, the arrestin and PY motifs, in Arn1 function (Fig. 5B,C). 3) Nakase et al. concluded that Any1 is required to store Aat1 in the Golgi and that deletion of *any1*^+^ mislocalizes the transporter to the PM (Nakase et al., 2013). By contrast, our findings revealed that Arn1 is required for Cat1 endocytosis in response to environmental conditions. These different actions of Arn1/Any1 on the distribution of Aat1 and Cat1, however, may be dependent on the feature of the amino acid transporters. In *S. cerevisiae*, two homologous ARTs, Aly1 and Aly2, have been known to express distinct actions for different amino acid transporters: Aly1 and Aly2 regulate intracellular sorting of Gap1, a general amino acid transporter, from endosome to the trans-Golgi network and/or the PM, but not its endocytosis (O'Donnell et al., 2010), whereas they also contribute to endocytosis of the aspartic acid/glutamic acid transporter Dip5 (Hatakeyama et al., 2010). In *S. pombe*, Aat1 is a homolog of Gap1, a general amino acid transporter (Nakase et al., 2010), whereas Cat1 is a specific transporter for cationic amino acids (Aspuria and Tamanoi, 2008). Therefore, similar to Aly1 and Aly2, Arn1/Any1 may also have distinct functions for different amino acid transporters. Further work is needed to understand how Arn1/Any1 affects different amino acid transporters.

Most recently, Zhao et al. have presented an interesting aspect of the arrestin function that ARTs together with Rsp5 compose a plasma membrane quality control system, which protects cells from proteotoxic stress in *S. cerevisiae* (Zhao et al., 2013). In *S. pombe* genome, there are at least 10 predicted ART genes including *arn1*^+^ and *arn2*^+^, whose products include an arrestin motif. Among these, for example, Ste7 appears to be involved in the mating pheromone signaling that is activated following activation of the heterotrimeric G-protein triggered by the interaction between pheromone peptides and those receptors, members in the G-protein-coupled receptor family (Matsuyama et al., 2000). Further studies are needed to gain the overview of functions of ARTs in *S. pombe*.

## MATERIALS AND METHODS

### Yeast strains, growth media, and general methods

*S. pombe* strains used in this study are listed in supplementary material Table S2. Cells were grown at 30°C, unless otherwise indicated, in yeast extract with supplements (YES) medium and Edinburgh minimal medium (EMM) supplemented with leucine or uracil, when indicated, which were prepared as described (Sabatinos and Forsburg, 2010). EMM-N, a nitrogen free version, was employed as a starvation medium. General and molecular genetic techniques followed standard protocols (Okazaki et al., 1990; Moreno et al., 1991). Disruption of *arn1*^+^ with the *ura4*^+^ cassette and integration of 3×HA-hphMX or -kanMX cassette at the C-terminal of *arn1*^+^ and *cat1*^+^ were performed using the PCR-based direct chromosomal integration methods (Bähler et al., 1998; Sato et al., 2005).

### Antibodies

Following antibodies were purchased from the commercial sources: anti-myc (9E10) and anti-α-Tubulin (B5-1-2) antibodies from Sigma (St Louis, MO); anti-HA (12CA5) and anti-GFP mouse antibodies from Roche (Mannheim, Germany); anti-ubiquitin (P4D1) from Cell Signaling Technology (Danvers, MA); horseradish peroxidase (HRP)-conjugated anti-mouse and anti-rabbit antibodies from Promega (Madison, WI).

### Genetic screening

JUp1211 cells were transformed with an *S. pombe* genomic library included in a high-copy plasmid, pTN-L1 (obtained from National BioResource Project (NBRP), Osaka Japan) and incubated overnight on EMM plate. Then, the cells were transferred on EMM plate containing 60 µg/ml canavanine (Sigma) by the replica plating method and further incubated. The recovered genomic plasmid clone from the cells was introduced again in JUp1211, and the transformants were assessed canavanine sensitivity. The recovered clone was sequenced to determine the included genomic region.

### Construction of expression plasmids and modified strains

To construct expression plasmids of *arn1*^+^, *atg14*^+^, and *arn2*^+^, the ORFs with approximately 600 bp of their 5′ and 3′ flanking regions were amplified by PCR using the genomic clone 1 plasmid or genomic DNA as a template and were cloned into pAL-KS vector (NBRP). To construct expression plasmids of *pub1*^+^ and its mutants, *pub1*^+^ ORF amplified by PCR using a cDNA library (NBRP) as a template was cloned into pREP41-2myc-6His vector, which was prepared from pREP41-2myc-6His-ubiquitin (kindly provided by Dr Nic Jones). Deletion of C2 and three WW domains in Pub1 was carried out by the PCR method. To construct expression plasmids of *cat1*^+^ and its point mutants, *cat1*^+^ ORF amplified by PCR using a genomic library (NBRP) as a template was cloned into pREP273-EGFP, which was generated by the replacement of multi-cloning sites together with the *nmt41* promoter (Basi et al., 1993) and 3HA fragment from pSLF273 (NBRP) and the insertion of EGFP ORF in *Not*I and *Bgl*II sites. The mutations of Cat1 were introduced by the site-directed mutagenesis.

To construct a series of the *arn1* mutant strains, *arn1*^+^ ORF and approximately 600 bp of its 5′ and 3′ flanking regions were cloned into pCR2.1. The point mutations in *arn1* were performed by the site-directed mutagenesis. The mutated *arn1* DNA fragments, 3HA-hphMX, and the *arn1*^+^ 3′-UTR were amplified and combined by PCR, and those fragments were integrated in the chromosomal locus of *arn1*^+^ by homologous recombination. A strain expressing Arn1-3EGFP was constructed by homologous recombination using pBS-*arn1*-*CT*-3EGFP-KanMX that the *arn1*-*CT* fragment replaced *ste20*-*CT* in the pBS-3EGFP-KanMX (kindly provided by Dr Kazuhiro Shiozaki). The sequences of all coding regions amplified by PCR were confirmed. To construct strains expressing the genomic copy of EGFP-fused Cat1-wt and its mutants (Cat1-a and -a-b-c), which are controlled under the *nmt41* promoter, JUp1211 cells were transformed with linearized pREP273-EGFP carrying wild-type Cat1 or its mutants, which was digested with *Mlu*I, to integrate into the *ars1*^+^ locus by homologous recombination.

### Protein preparation and phosphatase treatment

Protein extracts to assess Arn1-3HA was prepared from cells, which were boiled when harvested, as described previously (Nakashima et al., 2010). To assess Cat1 protein, cells, which were not boiled, were disrupted with glass beads in urea buffer (Nakashima et al., 2012) by vortexing twice for 5 minutes at 4°C, then mixed with 3× SDS sample buffer, and were incubated for 30 minutes at 37°C.

For the λ-phosphatase treatment of Cat1-GFP, cells were broken with glass beads in buffer A without NP-40 (Nakashima et al., 2010) by vortexing twice for 5 minutes at 4°C and were centrifuged at 14,000 *g* for 5 minutes at 4°C to prepare a membrane-rich fraction. The membrane-rich fraction was washed twice with buffer A without NP-40, and proteins were solubilized within urea buffer without sodium pyrophosphate, β-glycerophosphate, Na_3_VO_4_, *p*-nitrophenyl phosphate, and NaF for 30 minutes on ice. Glass beads and cell debris were removed by centrifugation. Supernatants including membrane proteins were diluted 7.5-fold into a reaction buffer and incubated with λ-phosphatase (400 IU; New England Biolabs, Ipswich, MA) for 30 minutes at 30°C.

### Immunoprecipitation and pull-down assay

To assess the interaction between Arn1-3HA and 2myc-6His-Pub1, cells were broken in buffer A with glass beads. After centrifugation at 12,000 *g* for 10 minutes and subsequently at 14,000 *g* for 20 minutes at 4°C, supernatants were incubated with anti-HA (3F10) affinity matrix (Roche) or with anti-myc (9E10) antibody and Protein-G-Sepharose (GE Healthcare Bio-Sciences, Buckinghamshire, England) for 2 hours at 4°C. Immunoprecipitates were washed three times with buffer A without protease inhibitors. Immunoprecipitated 2myc-6His-Pub1 was eluted with 600 µg/ml myc-peptides in buffer A.

To assess ubiquitination of immunoprecipitated-Arn1-3HA or -Cat1-3HA, cells were broken in urea buffer with glass beads, and cell extracts were diluted over 9-fold into buffer A. After centrifugation, supernatants were incubated with anti-HA (3F10) affinity matrix for 2 (Arn1-3HA) or 4 (Cat1-3HA) hours at 4°C. Immunoprecipitates were washed three times with buffer A without protease inhibitors.

For pull-down experiments for 6His-tagged ubiquitin, cells were broken in urea buffer without EDTA containing 10 mM imidazole with glass beads. After centrifugation, supernatants were incubated with Ni-NTA superflow (Qiagen, Venlo, The Netherlands) for 2 hours at 4°C, and the resins were washed four times with wash buffer [50 mM Tris-HCl (pH 7.5), 300 mM NaCl, 1% Triton X-100, and 20 mM imidazole]. Proteins were eluted with 300 mM imidazole in wash buffer.

### Immunoblotting

Protein extracts or immunoprecipitates were separated by SDS-PAGE, transferred to a nitrocellulose membrane, and immunoblotted with the indicated primary antibodies. Detection of proteins was performed using ECL plus system (GE Healthcare Bio-Sciences). When detecting ubiquitination of immunoprecipitated Cat1-3HA, a transferred membrane was dried and boiled in distilled water for 30 minutes before blocking (Swerdlow et al., 1986).

### Microscopy

All cell images were captured using fluorescence microscopes (BZ-8000 or BZ-9000; Keyence, Osaka, Japan) with a Nikon Plan Apo 60× oil immersion objective lens (NA 1.40, Nikon, Tokyo, Japan). Images were adjusted for brightness and contrast, inverted using Adobe Photoshop. To assess incorporation of FM4-64 dye (Life Technologies, Carlsbad, CA), cells were labeled with FM4-64 as described (Kita et al., 2004). In brief, cells were labeled with 50 µM FM4-64 for 30 minutes on ice, and then replaced to fresh medium without the dye at 30°C. Five minutes later, cells were examined under the fluorescence microscope.

## Supplementary Material

Supplementary Material
