# Supplementary material for: Fission yeast arrestin-related trafficking adaptor, Arn1/Any1, is ubiquitinated by Pub1 E3 ligase and regulates endocytosis of Cat1 amino acid transporter
Source: Biol Open. 2014 May 29;3(6):542–52. doi: 10.1242/bio.20148367 (PMC4058089; doi:10.1242/bio.20148367)
Supplement: Supplementary Material [file supp_3_6_542__index.html]

Fission yeast arrestin-related trafficking adaptor, Arn1/Any1, is ubiquitinated by Pub1 E3 ligase and regulates endocytosis of Cat1 amino acid transporter — Fission yeast arrestin-related trafficking adaptor, Arn1/Any1, is ubiquitinated by Pub1 E3 ligase and regulates endocytosis of Cat1 amino acid transporter — Supplementary Material 

# Fission yeast arrestin-related trafficking adaptor, Arn1/Any1, is ubiquitinated by Pub1 E3 ligase and regulates endocytosis of Cat1 amino acid transporter

## bio.20148367 Supplementary Material

**Files in this Data Supplement:**

- Supplementary Material - Akio Nakashima et al. doi: 10.1242/bio.20148367
