# Supplementary material for: Fission yeast arrestin-related trafficking adaptor, Arn1/Any1, is ubiquitinated by Pub1 E3 ligase and regulates endocytosis of Cat1 amino acid transporter
Source: Biol Open. 2014 May 29;3(6):542–52. doi: 10.1242/bio.20148367 (PMC4058089; doi:10.1242/bio.20148367)
Supplement: Supplementary Material [file supp_bio.20148367_bio.20148367-s1.pdf]

Biology Open (2014) 000, 1–11 doi:10.1242/bio.20148367

Supplementary Material

Akio Nakashima et al. doi: 10.1242/bio.20148367

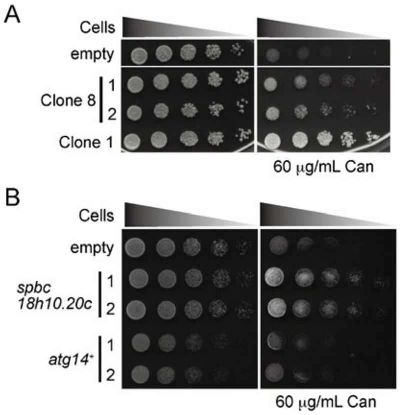

Fig. S1. Identification of *arn1*$^{+}$ as a gene resulting in resistance to canavanine in a genetic screening. (A) JUp1211 cells carrying an empty vector, the genomic clone 8 or clone 1 plasmid were spotted on EMM with or without 60 μg/ml canavanine and incubated for 3 days. (B) JUp1211 cells carrying an empty vector, pSLF173-*spbc18h10.20c*, or pREP1-*atg14*$^{+}$ were spotted on EMM with or without 60 μg/ml canavanine. Cells were incubated for 3 days (EMM) or 5 days (EMM with canavanine).

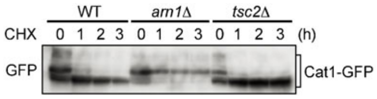

Fig. S3. Cat1 phosphorylation during the treatment with cycloheximide. PJ390 (WT), AN0223 (*arn1*Δ), and PJ380 (*tsc2*Δ) were grown in EMM and then treated with 30 μg/ml cycloheximide for the indicated times. Proteins were probed with the indicated antibody.

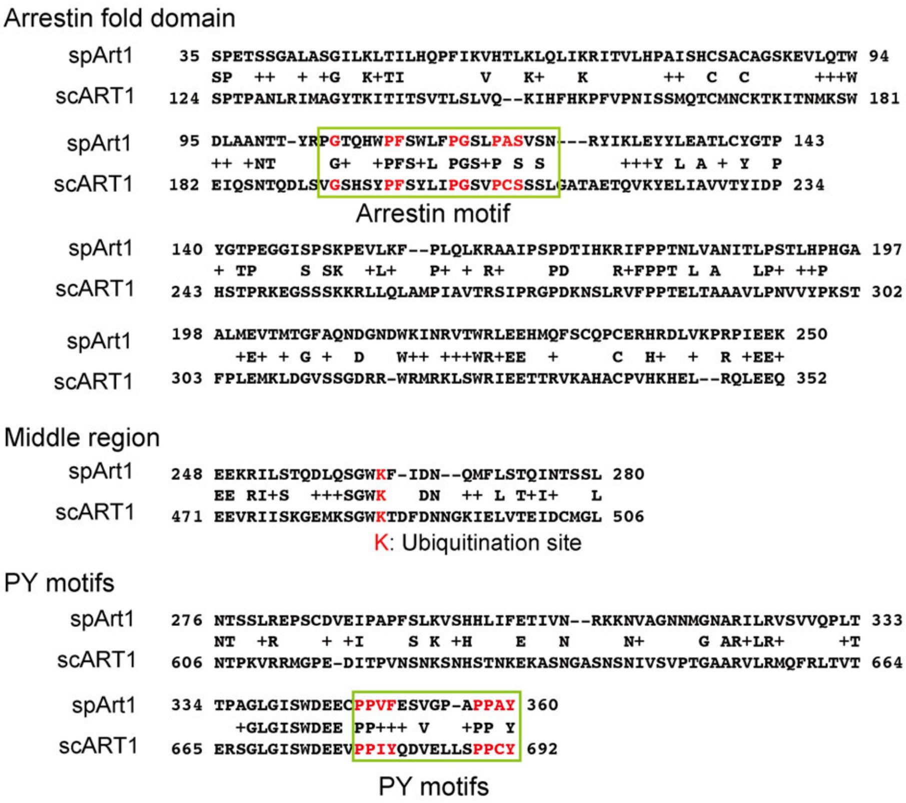

Fig. S2. Alignment of the predicted amino acid sequences of the conserved regions of *S. pombe* Arn1 and *S. cerevisiae* ART1. Sequence alignments were obtained from the BLAST program. Plus (+) represents the conservation substitutions. The conserved amino acid residues in the arrestin and PY motifs (enclosed green-line of squares) and of the ubiquitin site are denoted as red characters.

Biology Open

Biology Open (2014) 000, 1–11 doi:10.1242/bio.20148367

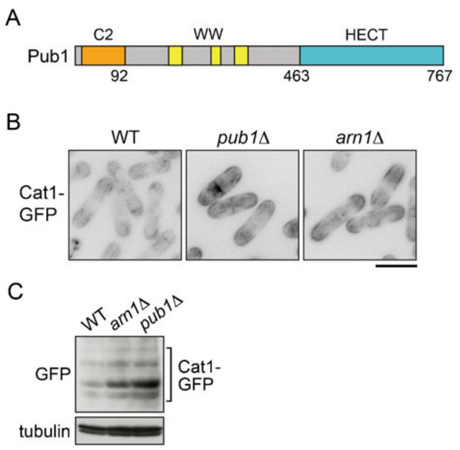

Fig. S4. Pub1 is involved in Cat1 function. (A) Schematic diagram of Pub1 that contains a C2 domain (orange box), three WW domains (yellow boxes), and a HECT domain (blue box). (B) AN0269 (WT), AN0223 (*arn1*Δ), and AN0280 (*pub1*Δ) were grown in EMM. The GFP images are shown inverted for clarity. (C) AN0269 (WT), AN0223 (*arn1*Δ), and AN0280 (*pub1*Δ) were grown in EMM. Proteins were probed with the indicated antibodies. Scale bar: 10 µm.

Table S1. Open reading frames included in genomic clones 1 and 8

Clone 1		Clone 8	
Chromosome II, 1803241–1810836		Chromosome I, 1595692–1605786	
SPNCRNA.377	non-coding RNA	srp102+	signal recognition particle receptor beta subunit Srp102, included its C-terminal region
SPBC18H10.19	human UVRAG (UV radiation resistance)/ Atg14 homolog	gln1+	Glutamate–ammonia lgase Gln1
SPBC18H10.20c	conserved fungal protein	SPAC23H4.05c	sequence orphan
		SPAC23H4.04	tRNA(5-methylaminomethyl-2-thiouridylate)-methyltransferase
		erv25+	COPII-coated vesicle component Erv25 (predicted)
		ppk9+	a serine/threonine protein kinase, included its C-terminal region

Biology Open

Biology Open (2014) 000, 1–11 doi:10.1242/bio.20148367

Table S2. ***S. pombe*** **strains used in this study**

Strain	Genotype	Reference/Source
L968	h^{90}	National-Bio-Resource-Project
L972	h^{-}	National-Bio-Resource-Project
JUp1204	h^{90} FY155	S. Forsburg (University of Southern California)
JUp1209	h^{90} $leu1^{-}$ ura4-D18	Lab stock
JUp1211	h^{90} $leu1^{-}$	Lab stock
PJ001	h^{-} tsc2::kanMX	Aspuria and Tamanoi, 2008
PJ380	h^{-} tsc2::kanMX $cat1^{+}$-GFP-hphMX	Aspuria and Tamanoi, 2008
PJ390	h^{-} $cat1^{+}$-GFP-hphMX	Lab stock; Aspuria and Tamanoi, 2008
AN0194	h^{90} $leu1^{-}$ ura4-D18 arn1::$ura4^{+}$	This study
AN0195	h^{-} $leu1^{-}$ ura4-D18 arn1::$ura4^{+}$	This study
AN0196	h^{90} ura4-D18 arn1::$ura4^{+}$	This study
AN0207	h^{90} $leu1^{-}$ tsc2::kanMX	This study
AN0208	h^{90} $leu1^{-}$ ura4-D18 tsc2::kanMX arn1::$ura4^{+}$	This study
AN0209	h^{90} ura4-D18 tsc2::kanMX arn1::$ura4^{+}$	This study
AN0220	h^{-} $arn1^{+}$-3HA-hphMX	This study
AN0223	h^{-} ura4-D18 $cat1^{+}$-GFP-hphMX arn1::$ura4^{+}$	This study
AN0224	h^{-} ura4-D18 $cat1^{+}$-GFP-hphMX arn1::$ura4^{+}$ tsc2::kanMX	This study
AN0259	h^{+} pub1::hphMX $arn1^{+}$-3HA-kanMX	This study
AN0264	h^{-} $arn1^{K263R}$-3HA-hphMX	This study
AN0267	h^{-} $arn1^{P348F350P358Y360/4A}$-3HA-hphMX	This study
AN0269	h^{-} $cat1^{+}$-GFP-kanMX	This study
AN0273	h^{-} $arn1^{P116G117/WW}$-3HA-hphMX	This study
AN0276	h^{-} $leu1^{-}$ $cat1^{+}$-GFP-hphMX	This study
AN0280	h^{-} pub1::hphMX $cat1^{+}$-GFP-kanMX	This study
AN0282	h^{-} $arn1^{+}$-3HA-hphMX $cat1^{+}$-GFP-kanMX	This study
AN0284	h^{-} $arn1^{P348F350P358Y360/4A}$-3HA-hphMX $cat1^{+}$-GFP-kanMX	This study
AN0286	h^{-} $arn1^{P116G117/WW}$-3HA-hphMX $cat1^{+}$-GFP-kanMX	This study
AN0288	h^{-} $arn1^{K263R}$-3HA-hphMX $cat1^{+}$-GFP-kanMX	This study
AN0290	h^{-} $leu1^{-}$ pub1::hphMX $cat1^{+}$-GFP-kanMX	This study
AN0293	h^{90} $leu1^{-}$ $arn1^{+}$-3HA-hphMX	This study
AN0295	h^{90} $leu1^{-}$ $arn1^{K263R}$-3HA-hphMX	This study
AN0297	h^{90} $leu1^{-}$ $arn1^{P348F350P358Y360/4A}$-3HA-hphMX	This study
AN0319	h^{-} $leu1^{-}$ ura4-D18 $cat1^{+}$-GFP-hphMX arn1::$ura4^{+}$	This study
AN0330	h^{90} $cat1^{+}$-3HA-hphMX	This study
AN0335	h^{90} ura4-D18 arn1::$ura4^{+}$ $cat1^{+}$-3HA-hphMX	This study
AN0351	h^{90} $arn1^{+}$-3EGFP-kanMX-pBS-KS	This study
AN0352	h^{-} $arn1^{+}$-3EGFP-kanMX-pBS-KS	This study
AN0399	h^{90} pub1::hphMX $arn1^{+}$-3EGFP-kanMX-pBS-KS	This study
